# 

**Published:** 1877-07

**Authors:** 


☞ Read Notice of this Journal among advertisements.

WHOLE NUMBER, CCCLXXXVI. | July, 1877. | VOL. XXXV. NUMBER 1.

THE

### CHICAGO MEDICAL Journal and Examiner

**EDITOR:**

WILLIAM H. BYFORD, A.M., M.D.

**ASSOCIATE EDITORS:**

JAMES H. ETHERIDGE, M.D.
NORMAN BRIDGE, M.D.
JAS. NEVINS HYDE, A.M., M.D.
FERD. C. HOTZ, M.D.

**ASSISTANT EDITOR:**

E. FLETCHER INGALS, M.D.

CHICAGO MEDICAL PRESS ASSOCIATION.

AUG. 2D

1875.

CHICAGO:
*PUBLISHED BY CHICAGO MEDICAL PRESS ASSOCIATION,*
188 CLARK STREET.

**Published Monthly. Terms—Four Dollars per annum, IN ADVANCE.**
**Single Copies, Thirty-Five Cents.**

**Subscriptions and Communications should be sent to the Assistant Editor, E. F. INGALS, M.D., 188 Clark Street.**

